# Supplementary material for: Survival is reduced when endogenous period deviates from 24 h in a non-human primate, supporting the circadian resonance theory
Source: Sci Rep. 2020 Oct 22;10:18002. doi: 10.1038/s41598-020-75068-8 (PMC7582969; doi:10.1038/s41598-020-75068-8)
Supplement: Supplementary file 1 — Supplementary Information [file 41598_2020_75068_MOESM1_ESM.pdf]

Supplementary Information

Survival is reduced when endogenous period deviates from 24h in a non-human primate, supporting the circadian resonance theory

Clara Hozer[1], Martine Perret[1], Samuel Pavard[2], Fabien Pifferi[1*]

1. Unité Mécanismes Adaptatifs et Evolution, Muséum National d'Histoire Naturelle, CNRS, 1 avenue du Petit Château, 91800 Brunoy, France

2 Unité Eco-anthropologie (EA), Muséum National d'Histoire Naturelle, CNRS, Université de Paris, F-75016, Paris, France

* corresponding author

Supplementary Table S1: Estimated effect sizes (± SD) and p-values for all variables retained in the selected models with all individuals.

Models retained	Dev.tau		Sex		Season		Dev.tau:Sex		Dev.tau:Season		Sex:Season		Dev.tau:Sex:Season		AIC
	β	p	β	p	β	p	β	p	β	p	β	p	β	p	
1	-0.8 ± 0.9	0.4	-1.4 ± 0.9	0.1	0.6 ± 0.9	0.6	1.8 ± 1.0	0.07.	0.8 ± 1.0	0.4	0.8 ± 1.1	0.4	-2.2 ± 1.1	0.05	752
2	-0.3 ± 0.7	0.7	-0.8 ± 0.5	0.08.	1.2 ± 0.5	**0.02 ***	1.2 ± 0.6	**0.05 ***	-	-	-	-	-1.2 ± 0.5	**0.01***	751

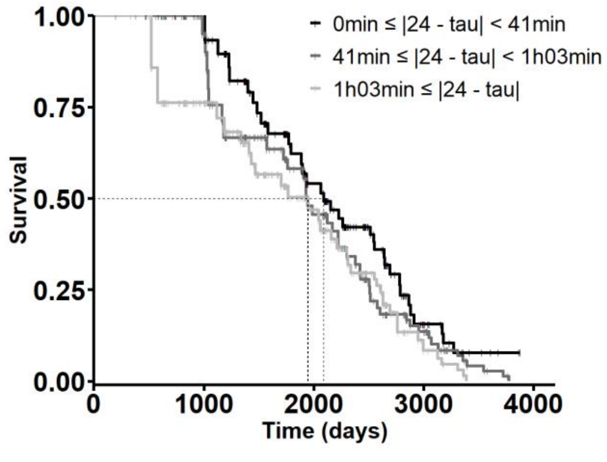

Supplementary Figure S1: Effect of Dev.tau on the survival of all individuals with increasing age. Individuals were distributed in tertiles to allow graphic representation, corresponding to specific cut-off of absolute deviation of *tau* from 24h. Dotted lines correspond to median survivals. Small solid vertical bars correspond to censored data.
